# Factors Associated With Discordance Between the Activated Partial Thromboplastin Time and Anti-factor Xa Activity During Extracorporeal Membrane Oxygenation: A Retrospective Cohort Study

**DOI:** 10.7759/cureus.114617

**Published:** 2026-08-16

**Authors:** Brian Simms, Carlos Carmona, Oliver Karam

**Affiliations:** 1 Pediatrics, Yale School of Medicine, New Haven, USA; 2 Pediatrics, Phoenix Children's Hospital, Phoenix, USA

**Keywords:** activated partial thromboplastin time, anticoagulation monitoring, anti-factor xa activity, assay discordance, critical care, extracorporeal membrane oxygenation support, unfractionated heparin

## Abstract

Background: Reliable anticoagulation monitoring during extracorporeal membrane oxygenation (ECMO) guides the balance between bleeding and thrombosis, yet the activated partial thromboplastin time (aPTT) and anti-factor Xa (anti-Xa) activity frequently disagree, and the factors associated with discordance are poorly defined. We sought to determine the prevalence of aPTT and anti-Xa discordance during unfractionated heparin anticoagulation on ECMO, to identify associated factors, and to characterize clinician response.

Methodology: We conducted a retrospective cohort study at a single ECMO center from 2019 to 2025, including neonates, children, and adults anticoagulated with heparin. aPTT and anti-Xa values drawn within six hours of each other were paired and classified as concordant or discordant. Generalized linear mixed-effects models with a random intercept for patient accounted for repeated measurements within patients.

Results: A total of 89 ECMO runs contributed 1,252 paired observations. Discordance occurred in 59.1% (740 of 1,252) of pairs, and the correlation between assays was moderate (Pearson R=0.41). Discordance was most frequent in neonates (464 of 671, 69.2%) compared with older children (32 of 66, 48.5%) and adults (244 of 515, 47.4%; unadjusted p<0.001). In univariate analysis, discordance was associated with lower weight and lower fibrinogen and with higher anti-Xa, aPTT, prothrombin time, and heparin dose. In mixed-effects models, a higher heparin infusion dose at the time of paired sampling was the only factor independently associated with discordance (adjusted odds ratio: 1.14; 95% CI: 1.07-1.22; p<0.001). Clinicians adjusted heparin after 396 of 740 discordant pairs (53.5%); changes aligned with anti-Xa in 329 of 740 (44.5%), with aPTT in 238 of 740 (32.2%), and with neither assay in 173 of 740 (23.4%). Anti-Xa-aligned changes were associated with a higher baseline heparin dose (adjusted odds ratio: 1.10; 95% CI: 1.02-1.19), whereas aPTT-aligned changes were associated with a higher aPTT (adjusted odds ratio: 1.04; 95% CI: 1.01-1.06) and higher fibrinogen (adjusted odds ratio: 1.01; 95% CI: 1.00-1.01).

Conclusions: Discordance between aPTT and anti-Xa is common during ECMO and varies by age group. Higher heparin infusion dose was independently associated with discordance, and clinician responses to discordant pairs were heterogeneous, highlighting persistent uncertainty in managing discordant anticoagulation assays.

## Introduction

Anticoagulation during extracorporeal membrane oxygenation (ECMO) requires balancing the prevention of circuit thrombosis against a high burden of hemorrhagic complications [1,2]. Unfractionated heparin remains the predominant anticoagulant, and its anticoagulant effect is monitored with the activated partial thromboplastin time (aPTT) and anti-factor Xa (anti-Xa) activity, two assays that reflect different components of heparin effect and can diverge because they are influenced by distinct clinical and analytic factors [2-4]. In pediatric ECMO, developmental hemostasis and evolving coagulation physiology further contribute to discordance between the two assays [2]. The aPTT is subject to variability with inflammatory states and factor perturbations, while anti-Xa remains susceptible to analytic interference from hyperbilirubinemia or plasma-free hemoglobin [2,3,5].

That the two assays disagree is well established in both pediatric and adult extracorporeal support cohorts [6,7]. The 2021 Extracorporeal Life Support Organization (ELSO) anticoagulation guideline acknowledges discrepant aPTT and anti-Xa results and outlines a practical approach to managing them [3]. What remains unknown is what drives that disagreement. Existing work has largely quantified how often discordance occurs rather than identified the conditions under which it arises, so it is not yet possible to say whether divergence reflects patient physiology, anticoagulation intensity, analytic interference, or the timing of sampling. Without that knowledge, guidance on interpreting conflicting results rests largely on consensus, and bedside responses remain heterogeneous [3,8,9]. 

We therefore designed this study around the determinants of discordance rather than its frequency. Our primary objective was to identify the clinical and laboratory factors independently associated with discordance between aPTT and anti-Xa, accounting for repeated measurements obtained within individual patients. Our secondary objectives were to determine the prevalence and patterns of discordance in this cohort, which provide the denominator against which those factors are interpretable, and to characterize how clinicians titrated heparin after a discordant pair, which indicates whether either assay is preferentially followed when the two conflict.

## Materials and methods

Study design and population

We performed a single-center retrospective cohort study at Yale New Haven Hospital in New Haven, Connecticut, including all patients supported with ECMO between July 2019 and June 2025. The study was conducted in accordance with the Declaration of Helsinki, and the Yale Human Research Protection Program approved the study with a waiver of informed consent (approval number: 2000038325; date: August 2024). The manuscript was prepared in accordance with the Strengthening the Reporting of Observational Studies in Epidemiology (STROBE) reporting guideline. 

Adult, pediatric, and neonatal patients were eligible if they received unfractionated heparin for anticoagulation and remained on ECMO support for at least 24 hours. Patients anticoagulated with alternative agents, such as bivalirudin or argatroban, and those who died within 24 hours of cannulation were excluded. For patients with multiple ECMO runs during the same hospitalization, only the first run was included to avoid repeated patient-level outcomes.

Data collection

Demographic and clinical variables were extracted from the electronic medical record and included age, weight, and ECMO configuration, either veno-arterial (VA) or veno-venous (VV). All aPTT and anti-Xa levels obtained during ECMO support were collected. When multiple eligible measurements were available, each aPTT value was paired with the temporally closest anti-Xa value within six hours, and each laboratory result was used in only one pair. Values reported by the laboratory as outside the limits of quantification were recorded at the limit for analysis: aPTT results reported as greater than 140 seconds were recorded as 140 seconds, and anti-Xa results reported as less than 0.1 IU/mL were recorded as 0.1 IU/mL.

For each paired laboratory assessment, the heparin infusion rate at the time of blood draw was recorded, along with any bolus administration. Changes in infusion rate within six hours following the paired assay were documented. The most recent platelet count, prothrombin time (PT), and fibrinogen level within the preceding 24 hours were also recorded and included as laboratory covariates.

Concordance definition

Therapeutic targets were based on institutional practice aligned with ELSO guidance, with an aPTT goal of 60-90 seconds and an anti-Xa goal of 0.3-0.7 IU/mL [3]. Concordance required both assays to fall into the same category, defined as low, therapeutic, or high, while discordance was defined as disagreement between assay categories. Major discordance specifically referred to one assay being below goal and the other above goal.

Heparin adjustment definition

Heparin was titrated by the bedside team toward institutional targets of an aPTT of 60-90 seconds and an anti-Xa of 0.3-0.7 IU/mL. No institutional protocol specified the frequency of aPTT or anti-Xa testing, and no protocol or algorithm directed management when the two assays disagreed. Testing frequency and the response to discordant results were therefore at clinician discretion throughout the study period.

Heparin dose adjustments, including boluses, were evaluated in the six-hour window following each paired laboratory measurement. An adjustment was considered appropriate for aPTT if the infusion rate was increased when the aPTT was below 60 seconds or decreased when above 90 seconds. Similarly, an adjustment was considered appropriate for anti-Xa if the infusion rate was increased when the anti-Xa level was below 0.3 IU/mL or decreased when above 0.7 IU/mL. For an assay value within the therapeutic range, no change in heparin infusion was considered aligned with that assay. Adjustments consistent with both assays were categorized as appropriate for both, whereas those inconsistent with either assay were classified as aligned with neither.

Statistical analysis

Continuous variables are reported as medians with interquartile ranges (IQRs) or means with standard deviations, as appropriate; categorical variables are summarized as counts with percentages. Because multiple paired laboratory measurements were obtained per patient, all regression analyses incorporated generalized linear mixed-effects models (GLMMs) with a random intercept to account for within-patient clustering. Given collinearity between age and weight, only weight was included in multivariable models. Adjusted odds ratios (aORs) with 95% confidence intervals (CIs) are reported, and the results of the GLMMs are presented as forest plots.

Prevalence of concordance and discordance was described using proportions of concordant, minor discordant, and major discordant assay pairs. The association between aPTT and anti-Xa values was evaluated using Pearson correlation. Factors associated with discordance were examined using mixed-effects logistic regression with discordance, coded as yes or no, as the dependent variable. Candidate predictors included ECMO mode, platelet count, PT, fibrinogen level, and heparin dose at the time of laboratory measurement, selected based on biological plausibility and prior literature. Clinician response was evaluated using mixed-effects logistic regression to identify variables associated with heparin dose adjustment within six hours of a paired assay. 

Laboratory values that were not obtained were treated as missing and were not imputed. As laboratory testing during ECMO was influenced by both protocolized practice and clinician discretion, missingness may have been related to patient condition or clinical decision-making. Because missingness was related to patient population and to discretionary rather than protocolized testing, imputation under a missing-at-random assumption was not considered defensible, and analyses were restricted to available complete cases, with the attendant potential for selection bias acknowledged. All analyses were performed using IBM SPSS Statistics for Windows, Version 31.0.1 (IBM Corp., Armonk, New York, United States), and a two-sided p-value of less than 0.05 was considered statistically significant.

## Results

Epidemiology of concordance and discordance

**Figure 1:**
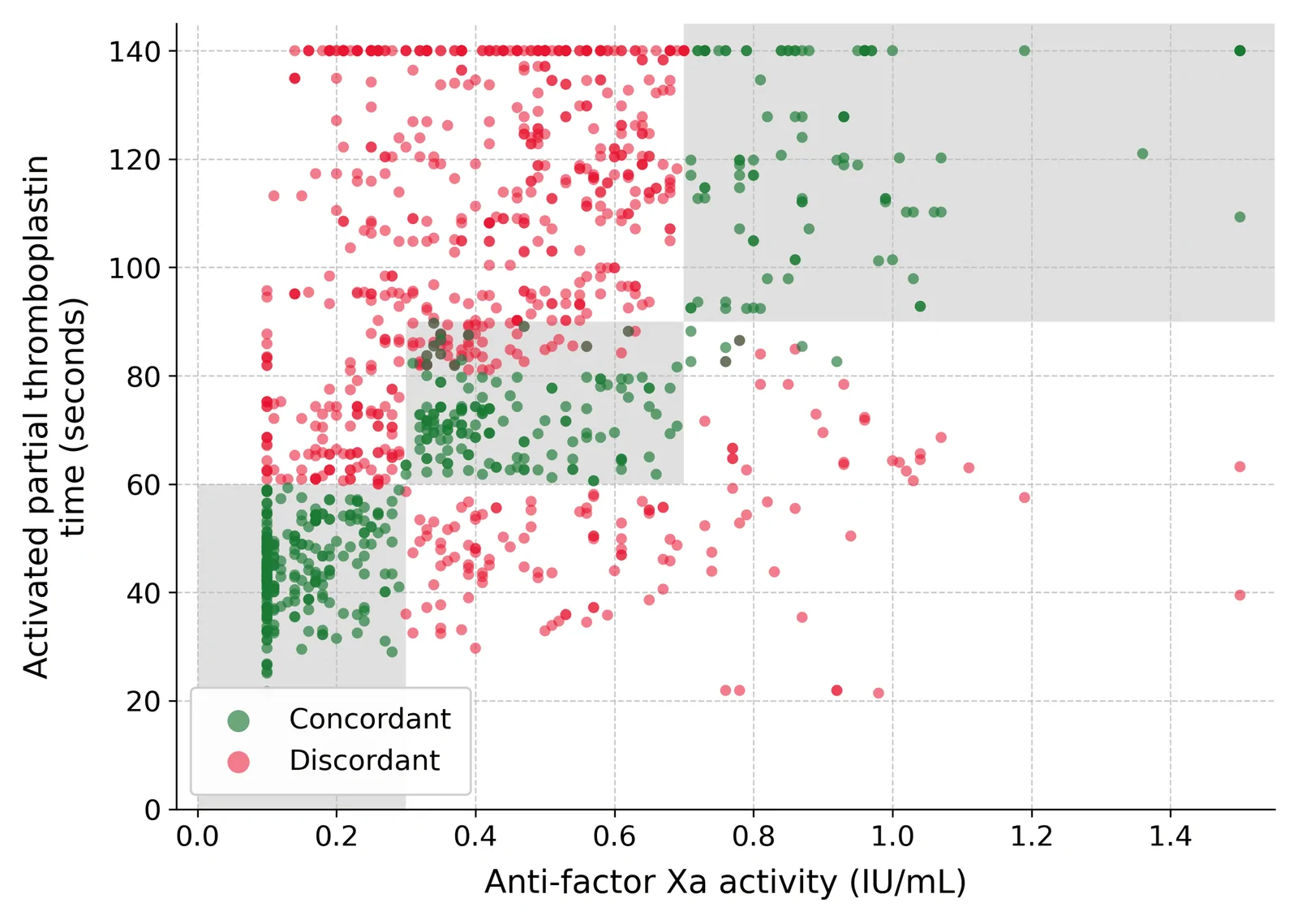
Scatter plot of paired anti-Xa and aPTT measurements obtained during extracorporeal membrane oxygenation aPTT: activated partial thromboplastin time; anti-Xa: anti-factor Xa Each point represents a single paired laboratory assessment (n=1,252). Green points indicate concordant assay pairs, defined as both values being low, within the therapeutic range (anti-Xa: 0.3-0.7 IU/mL; aPTT: 60-90 seconds), or high according to institutional targets. Red points indicate discordant assay pairs, defined as disagreement between anti-Xa and aPTT categories, either minor or major. Shaded regions denote therapeutic ranges for each assay. A moderate correlation was observed between assays (Pearson R=0.41), with substantial dispersion and frequent discordance across the spectrum of anticoagulation intensity. The horizontal axis shows anti-Xa activity in IU/mL, and the vertical axis shows the aPTT in seconds. The association between the two assays was assessed using Pearson correlation, and a two-sided p-value below 0.05 was considered statistically significant.

During the study period, 89 unique ECMO runs met the inclusion criteria, among which 12 (13.5%) were neonates, 10 (11.2%) were older children, and 67 (75.3%) were adults. The median weight was 82.1 kg (IQR: 54.7-104.3), and 55 of the 88 patients with a recorded configuration (62.5%) received VA ECMO. These patients contributed 1,252 paired observations of anti-Xa and aPTT obtained within a six-hour window, with a median of three paired observations each (IQR: 2-9). Discordance was observed in 59.1% (740 of 1,252) of paired samples, while concordance was observed in the remaining 40.9% (512 of 1,252) (Figure 1).

Discordance was more frequent in neonatal observations (464 of 671, 69.2%) than in observations from older children (32 of 66, 48.5%) or adults (244 of 515, 47.4%; p<0.001). Among discordant pairs, aPTT was in a higher therapeutic category than anti-Xa in 606 of 740 observations (81.9%), whereas anti-Xa was in a higher category in 134 observations (18.1%). The most frequent discordance pattern was a therapeutic anti-Xa with a high aPTT (392 of 740, 53%). The overall relationship between the two assays was moderate, with a Pearson R of 0.41.

Missingness

Complete data for all model covariates were available for 901 of 1,252 pairs (72%). Missingness was driven by fibrinogen, unavailable for 332 pairs (26.5%), and PT, unavailable for 173 pairs (13.8%). The platelet count was missing for six pairs (0.5%) and ECMO configuration for three (0.2%), and weight, heparin dose, and both assay values were complete. Missingness was strongly age-dependent: complete covariate data were available for 649 of 671 neonatal pairs (96.7%) and all 66 pairs from older children, but for only 186 of 515 adult pairs (36.1%).

Factors associated with discordance

In univariate comparisons restricted to the 901 pairs with complete covariate data (Table 1), discordant paired samples had lower weight (4.8 kg (IQR: 3.2-6.5) vs. 5.7 kg (IQR: 4.8-76.3); p<0.001), higher anti-Xa (0.45 IU/mL (IQR: 0.28-0.57) vs. 0.37 IU/mL (IQR: 0.17-0.71); p<0.001), higher aPTT (109 seconds (IQR: 85-134) vs. 69 seconds (IQR: 50-87); p<0.001), and higher PT (13.6 seconds (IQR: 12.4-15.3) vs. 12.5 seconds (IQR: 11.9-13.3); p<0.001). Discordant pairs also had lower fibrinogen (246 mg/dL (IQR: 201-284) vs. 281 mg/dL (IQR: 227-370); p<0.001) and a higher heparin dose (35 units/kg/hr (IQR: 23-48) vs. 28 units/kg/hr (IQR: 10.8-45); p<0.001).

**Table 1:** Laboratory characteristics and heparin dose of paired aPTT and anti-Xa observations, stratified by concordance status aPTT: activated partial thromboplastin time; anti-Xa: anti-factor Xa; PT: prothrombin time Values are median (25th-75th percentile). Weight is that of the patient contributing the pair and, like every other row, is summarized across pairs rather than across patients. All comparisons are at the level of the paired assay and are restricted to the 901 pairs with complete covariate data (346 concordant and 555 discordant); pairs missing a platelet count, PT, fibrinogen level, or extracorporeal membrane oxygenation configuration were excluded. P-values were derived from generalized linear mixed-effects models with a random intercept for patient, accounting for repeated observations within patients. An asterisk (*) denotes a p-value that is statistically significant at a two-sided threshold of 0.05.

Variable	Concordance	Discordance	P-value
Weight (kg)	5.7 (4.8-76.3)	4.8 (3.2-6.5)	<0.001*
Anti-Xa (IU/mL)	0.37 (0.17-0.71)	0.45 (0.28-0.57)	<0.001*
aPTT (seconds)	69 (50-87)	109 (85-134)	<0.001*
Platelet count (×10⁹/L)	80 (57-114)	83 (63-111)	0.10
PT (seconds)	12.5 (11.9-13.3)	13.6 (12.4-15.3)	<0.001*
Fibrinogen (mg/dL)	281 (227-370)	246 (201-284)	<0.001*
Heparin dose (units/kg/hr)	28 (10.8-45)	35 (23-48)	<0.001*

**Figure 2:**
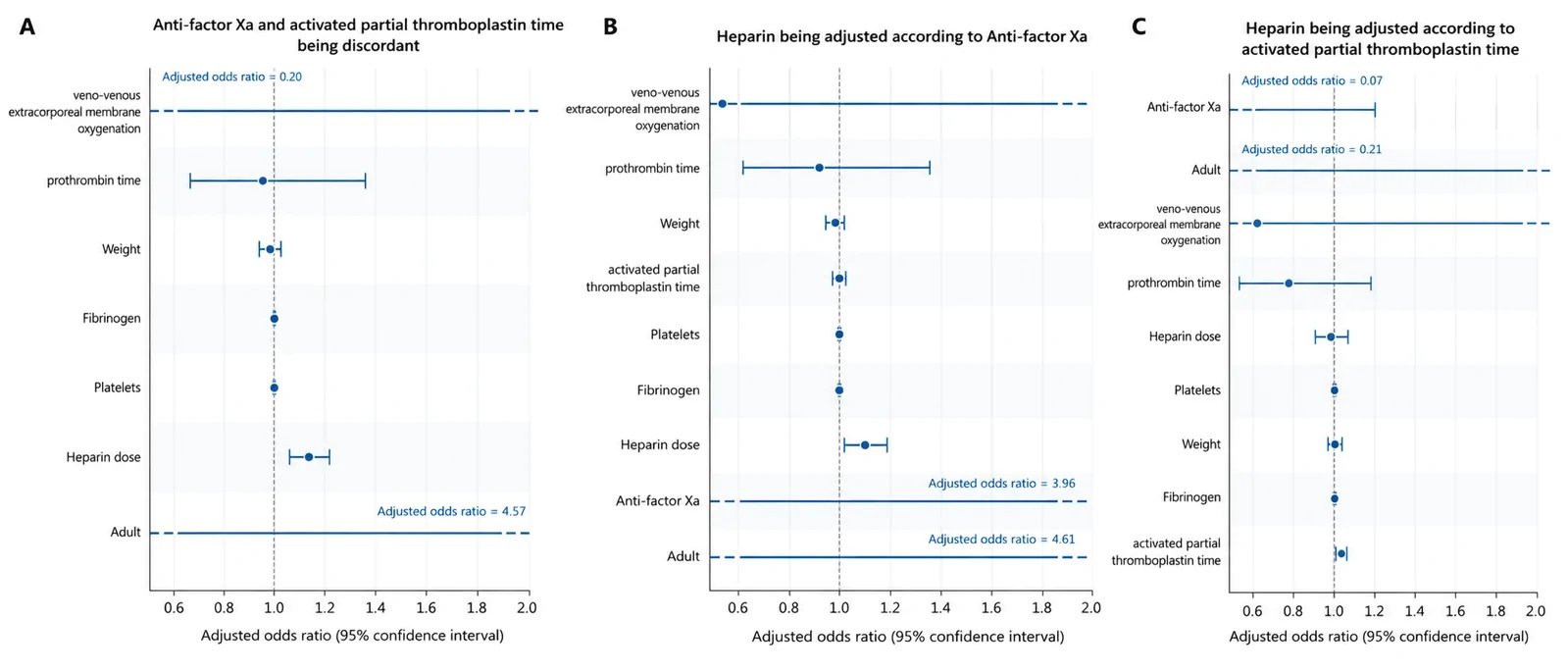
Forest plots of generalized linear mixed-effects models of assay discordance and of subsequent heparin adjustment aPTT: activated partial thromboplastin time; anti-Xa: anti-factor Xa; aOR: adjusted odds ratio All generalized linear mixed-effects models include a random intercept for patient. Variables are ordered from lowest to highest aOR. (A) Factors associated with discordance between anti-Xa and aPTT among all paired observations (n=1,252). (B) Factors associated with subsequent heparin adjustment being aligned with the anti-Xa value, restricted to discordant pairs (n=740). (C) Factors associated with subsequent heparin adjustment being aligned with the aPTT value, restricted to discordant pairs (n=740). The dashed vertical line indicates the null value of 1.0. Dashed segments at the end of a confidence interval bar indicate that the bar extends beyond the displayed range; italicized labels indicate point estimates that fall outside the displayed range. Odds ratios are unitless. A two-sided p-value below 0.05 was considered statistically significant.

In GLMMs accounting for repeated measures, heparin infusion dose at the time of paired laboratory collection emerged as the only independent factor associated with discordance, with higher dosing intensity associated with increased odds of discordant categorization of aPTT and anti-Xa (aOR: 1.14; 95% CI: 1.07-1.22; p<0.001). No other candidate predictor was independently associated with discordance, including ECMO mode, weight, platelet count, PT, and fibrinogen level (Figure 2A).

Association with heparin adjustment and anticoagulation intensity

The time-weighted average heparin infusion rate across all ECMO runs was 12.24 units/kg/hr, whereas the median infusion rate recorded at the time of paired laboratory collection was 25.0 units/kg/hr (IQR: 13.0-42.0). At the time of paired testing, discordant pairs were associated with higher heparin doses compared with concordant pairs (median: 35 units/kg/hr (IQR: 23-48) vs. 28 units/kg/hr (IQR: 10.8-45); p<0.001). Minor discordance accounted for 613 of 740 discordant pairs (82.8%) and major discordance for 127 of 740 (17.2%). The median heparin dose was higher with minor than with major discordance (30.0 units/kg/hr (IQR 19.2-45.0) vs. 22.0 units/kg/hr (IQR: 10.1-28.0); p<0.001).

Following concordant pairs, heparin management was classified as appropriate for both assays in 42% (215 of 512) of instances, meaning that subsequent changes in heparin were not aligned with concordant assay targets in 58% (297 of 512). Following discordant pairs, clinicians adjusted the heparin dose in 53.5% (396 of 740). Across all discordant pairs, post-pair heparin changes aligned with anti-Xa in 44.5% (329 of 740), aligned with aPTT in 32.2% (238 of 740), and aligned with neither assay in 23.4% (173 of 740).

In mixed-effects models of heparin changes, the likelihood of anti-Xa-aligned adjustments was associated with a higher baseline heparin dose (aOR: 1.10; 95% CI: 1.02-1.19; p=0.013) (Figure 2B). The likelihood of aPTT-aligned adjustments was associated with higher fibrinogen (aOR: 1.01; 95% CI: 1.00-1.01; p=0.014) and with a higher aPTT (aOR: 1.04; 95% CI: 1.01-1.06; p=0.006) (Figure 2C).

## Discussion

In this single-center retrospective cohort study of 89 ECMO runs, discordance between aPTT and anti-Xa was strikingly common, occurring in more than half the paired observations, with only moderate correlation between assays. The clinical significance of these findings lies less in the frequency of discordance than in its determinants and in how those determinants should inform the interpretation of and response to discordant assays during ECMO. Discordance varied significantly by age group, being more prevalent in neonates than in older children and adults. In univariate analysis, discordant pairs were associated with lower weight, higher anti-Xa, higher aPTT, higher PT, lower fibrinogen, and higher heparin dose. However, in GLMMs accounting for repeated measures, heparin infusion dose was the only independent factor associated with discordance, suggesting that most univariate associations reflect confounding by anticoagulation intensity rather than independent biologic drivers. Clinician heparin management following discordant results was heterogeneous: dose adjustments were made after only half of discordant pairs, and of all discordant pairs, adjustments aligned with anti-Xa in nearly half the cases and with aPTT in about a third of the cases. Clinicians were more likely to make anti-Xa-aligned adjustments when baseline heparin doses were higher and more likely to follow aPTT when aPTT and fibrinogen values were higher.

Our finding that laboratory discordance is highly prevalent is consistent with recent extracorporeal support literature and underscores the complexity of monitoring unfractionated heparin during ECMO [4,6]. These assays capture different aspects of coagulation: anti-Xa reflects functional heparin activity through factor Xa inhibition, whereas aPTT is a clot-based measure reflecting intrinsic and common pathway activity and is sensitive to coagulation factor levels and inflammatory shifts [2,3]. Systematic reviews highlight substantial variability in monitoring strategies across centers, reflecting ongoing uncertainty regarding optimal anticoagulation monitoring during ECMO [10]. As a result, isolated laboratory values may not fully reflect overall anticoagulation status and should be interpreted within the broader clinical and laboratory context, especially when multiple assays are followed simultaneously and provide discordant signals [3].

A key finding from our analysis is the assessment of factors contributing to discordance. In the univariate analyses, lower weight, higher anti-Xa, higher aPTT, higher PT, lower fibrinogen, and higher heparin dose were associated with discordance, whereas heparin dose was the sole independent factor associated with discordance. This multivariable shift suggests that many apparent univariate relationships may reflect confounding or covariance with anticoagulation intensity rather than independent biologic drivers [11]. Heparin dose is therefore best understood as the variable that remained associated with discordance after adjustment rather than as its cause, since the retrospective design and the possibility of residual confounding preclude causal inference.

The following mechanistic explanations are hypothesis-generating. Our data identify an association between dosing intensity and discordance but contain no direct measure of antithrombin activity, factor VIII, plasma-free hemoglobin, or inflammatory markers and cannot adjudicate among these candidate pathways. One possible explanation is that, at higher dosing intensities, small differences in assay sensitivity to non-heparin influences may become more apparent, increasing the likelihood of discordance even when samples are obtained within a narrow time window. This may be particularly relevant to aPTT, which does not rise proportionally across the full range of unfractionated heparin exposure and may demonstrate nonlinear behavior or plateauing at higher heparin concentrations [12-15]. High-dose scenarios may also reflect some degree of heparin resistance physiology, including antithrombin deficiency, increased heparin binding, enhanced clearance, or elevations in factor VIII and fibrinogen, which can blunt the aPTT response despite continued heparin exposure [5,13,14]. This pattern, often driven by acute-phase elevations in factor VIII and fibrinogen, has been described as pseudo-heparin resistance, in which aPTT may appear subtherapeutic despite sufficient anti-Xa activity, potentially prompting heparin escalation and contributing to assay discordance [16]. Thus, higher heparin dose may not directly cause discordance, but may identify patients in whom nonlinear aPTT behavior, ceiling effects, and altered heparin responsiveness make divergence from anti-Xa more likely.

We also observed heterogeneity in heparin adjustment following discordant paired results. Among discordant pairs, heparin dose adjustments aligned with anti-Xa in 329 of 740 (44.5%), aligned with aPTT in 238 of 740 (32.2%), and aligned with neither assay in 173 of 740 (23.4%). In mixed-effects models of clinician response, anti-Xa-aligned adjustments were associated with a higher baseline heparin dose, consistent with evidence that anti-Xa correlates more closely with heparin dose than the aPTT in pediatric ECMO, although the aPTT has been reported to trend more reliably in other pediatric cohorts [17,18]. In contrast, aPTT-aligned adjustments were associated with higher aPTT and higher fibrinogen. Because fibrinogen is also an acute-phase protein that increases in systemic inflammation, these findings may suggest that dosing intensity and inflammatory context influence which assay clinicians follow when values conflict [16]. These findings are clinically relevant because discordant high anti-Xa or high aPTT values may prompt heparin reduction to limit perceived bleeding risk, whereas low values may prompt dose escalation, yet clinicians did not respond uniformly when assays conflicted. The association between higher fibrinogen and aPTT-aligned changes may indicate that broader coagulation context influenced bedside heparin titration, although this requires further study.

Current extracorporeal guidance acknowledges assay limitations but remains largely consensus-driven, reinforcing the need to link discordance response strategies to clinical outcomes [3,19]. Given the potential influence of inflammatory context on clot-based assays, future prospective multicenter studies should evaluate guideline-informed discordance response pathways, including approaches that account for dosing intensity and inflammatory markers, and examine associations with bleeding, thrombosis, transfusion burden, and heparin dose variability [3,10].

Limitations

This study has limitations inherent to retrospective, single-center analyses. Discordance classification is sensitive to exact measurement timing and concurrent interventions, and the six-hour pairing window may allow changes in patient status between measurements [3]. However, the median time between paired measurements was 0.4 hours, limiting this misclassification. Separately, clinician response was inferred from heparin infusion changes within six hours of a paired result, and this attribution may be imperfect. A dose change in that window could have been prompted by a subsequent laboratory value, by a bleeding or thrombotic event, by a circuit intervention, or by a procedural hold rather than by the paired result itself, and conversely, a decision attributable to the paired result could have been enacted outside the window. Alignment categories should therefore be read as describing the observed pattern of titration following discordance rather than as a direct measure of clinician intent. Both assays are subject to the limits of quantification, and results beyond those limits are not reported as exact values; 172 of the 1,252 aPTT values were reported as greater than 140 seconds and 136 anti-Xa values as less than 0.1 IU/mL, together affecting 308 pairs. Category assignment was unaffected, because every censored aPTT value exceeded the high threshold and every censored anti-Xa value fell below the therapeutic threshold regardless of the true result, but the median values reported in Table 1 and the continuous assay terms in the mixed-effects models treat a substituted limit as an observation. Of note, both assays were performed in the hospital clinical laboratory, but the analyzers and reagents used for the aPTT and the anti-Xa assay changed on multiple occasions over the six-year study period, and we were unable to reconstruct which platform and reagent applied to each paired measurement. Because reagent responsiveness to heparin differs across aPTT reagents in particular, some of the observed discordance may reflect assay platform rather than patient physiology, and our results may not transfer directly to centers using a single fixed assay configuration. Paired observations were also distributed unevenly across age groups. Although adults accounted for 67 of the 89 runs, neonates and older children contributed 737 of the 1,252 paired observations, so weight measured at the level of the paired assay reflects the sampling distribution rather than the composition of the cohort. One possible explanation for the association between lower weight and discordance is therefore that neonates and older children were sampled more frequently than adults, rather than an effect of patient size itself. Furthermore, the older child stratum contributed only 10 runs and 66 paired observations, so age group comparisons involving this group are imprecise and should be regarded as descriptive. Although mixed-effects modeling accounted for repeated measures and numerous confounding variables, unmeasured confounders remain, such as markers of inflammation or plasma-free hemoglobin. Therefore, heparin dose may partly serve as a marker for these unmeasured drivers rather than acting as a direct cause of discordance. Because the mechanisms proposed above, including nonlinear aPTT behavior and heparin resistance physiology, were not measured in this cohort, prospective studies incorporating antithrombin activity, factor VIII, plasma-free hemoglobin, and inflammatory markers are required to test them. In addition, complete-case analysis restricted the covariate-adjusted models to 901 pairs contributed by 45 of the 89 patients, and because fibrinogen and PT were far less often available in adults, that analytic set is more heavily neonatal than the full cohort (72% vs. 53.6% of pairs). The adjusted estimates therefore describe the population in which complete coagulation panels were obtained, and selection bias cannot be excluded. We did not directly compare clinical outcomes across alignment strategies; therefore, our results define factors associated with discordance and describe clinician response patterns rather than establishing superiority of any monitoring approach.

## Conclusions

In this single-center ECMO cohort, discordance between the aPTT and anti-Xa activity was common, occurring in more than half of paired observations, and was more frequent in neonates than in older children or adults. Higher heparin infusion dose was the only factor independently associated with discordance after accounting for repeated measurements within patients, which suggests that the associations seen on univariate testing largely reflect anticoagulation intensity rather than independent biologic drivers. Higher dosing intensity may therefore identify patients in whom nonlinear behavior of the aPTT and altered heparin responsiveness make divergence from anti-Xa activity more likely, although these mechanisms were not measured in this cohort and remain hypotheses to be tested prospectively.

Clinician response to discordant results was heterogeneous. Heparin was adjusted after only about half of discordant pairs, and when it was adjusted, changes followed anti-Xa activity in some instances, the aPTT in others, and neither assay in nearly a quarter. Heparin management also differed by discordance pattern, with anti-Xa-aligned changes associated with a higher baseline heparin dose and aPTT-aligned changes associated with higher values of that assay and higher fibrinogen. This heterogeneity underscores persistent uncertainty regarding the optimal approach to interpreting and managing discordant anticoagulation assays. Prospective studies are needed to determine whether a standardized approach to interpreting discordant results improves bleeding, thrombosis, and other patient-centered outcomes during ECMO.
